# Supplementary material for: Incidence and prevalence of hypertension among HIV-TB co-infected participants accessing treatment: A five-year prospective cohort analysis
Source: PLoS One. 2024 Jan 29;19(1):e0297224. doi: 10.1371/journal.pone.0297224 (PMC10824407; doi:10.1371/journal.pone.0297224)
Supplement: Attachment — Submitted filename: PLoS One Reviewer reoprt.docx [file pone.0297224.s001.docx]

**PLoS One Reviewer’s report**

**General remarks**

Congratulations to the authors for this piece of work highlighting the growing burden of cardiovascular risk conditions in PLWHA co-infected with TB. Findings from this study would help to guide appropriate intervention for early detection and treatment of hypertension in PLWHA.

General comments

Participants for this study were HIV/TB coinfected. I would suggest this to be emphasised in the title.

The authors used antiretroviral treatment and antiretroviral therapy interchangeably. I suggest using antiretroviral therapy throughout the abstract and the main text

All abbreviations in the abstract and main text should be defined on first appearance in the text.

**Specific comments**

Methods

Line 137: “The start of the ART initiation in the SAPiT study was taken as baseline” – Do the author mean “ART initiation date was taken as baseline”.

Lines 149 – 151: Procedures for blood pressure measurements need to be explained in detail.

Specify guidelines did you use for BP measurement. Specify the model of automated BP sphygmomanometer, weight scale and stadiometer

Lines 156-157: Specify guidelines used to define hypertension

Lines 160 - 162: Definition for hypertension does not reflect the cited guideline (JNC8) i.e., “systolic measurements above 139 mmHg and/or diastolic measurements above 89 mmHg on two

162 consecutive visits or current use of antihypertensive therapy”

Lines 162 – 163: The sentence “Patients already on medication for hypertension were considered to be hypertensive” is a repetition of the information already included in the definition for hypertension. Consider deleting this sentence of rephrase the definition.

Data analysis

The authors chose to define hypertension occurring during the first 3 months post enrolment as prevalent hypertension. What was the basis to choose 3 months’ time-frame in the definition?

The authors considered BMI as a time varying covariate in the multivariable logistic regression model but did not allow BMI as a time varying co-variate in the Poisson regression model. Would be helpful to the reader to understand why BMI was not treated as time varying co-variate in the Poisson model.

Results

642 participants were followed-up but the results are available for 507 participants. What was the reason for not including 135 participants? How do the 135 participants not included in the analysis compare with the remaining 507 in terms of baseline characteristics?

Line 278 – 282: comparison of adjusted Hazard Ratios (aHRs) should mention the reference group indicated in table 3

Table 2

- HPT should be defined on the table footer
- Variables with missing values should be indicated

Table 3

- How many participants were included in the regression analysis?
- All abbreviations (i.e., BP, CI) should be defined on the table footer. The symbol § should appear on in all HR abbreviations

Table 4

- How many participants were included in the regression analysis?
- All abbreviations (i.e., BP, CI etc.) needs to be defined on the table footer
